# Supplementary material for: Identification of Freezing-Responsive microRNAs and Their Targets in Chinese Jujube by Small RNA and Degradome Sequencing
Source: Int J Mol Sci. 2026 Sep 4;27(17):7912. doi: 10.3390/ijms27177912 (PMC13566890; doi:10.3390/ijms27177912)
Supplement: Supplementary file 1 [file ijms-27-07912-s001.zip › Supplementary Material S2/novel_miR_45.pdf]

Provisional ID	:	novel_miR_45
Score total	:	0.3
Score for star read(s)	:	-1.3
Score for read counts	:	0
Score for mfe	:	0
Score for randfold	:	1.6
Score for cons. seed	:	
Total read count	:	104
Mature read count	:	104
Loop read count	:	0
Star read count	:	0

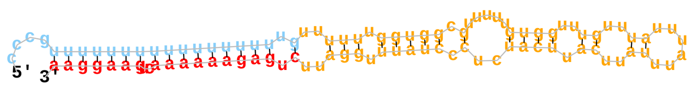

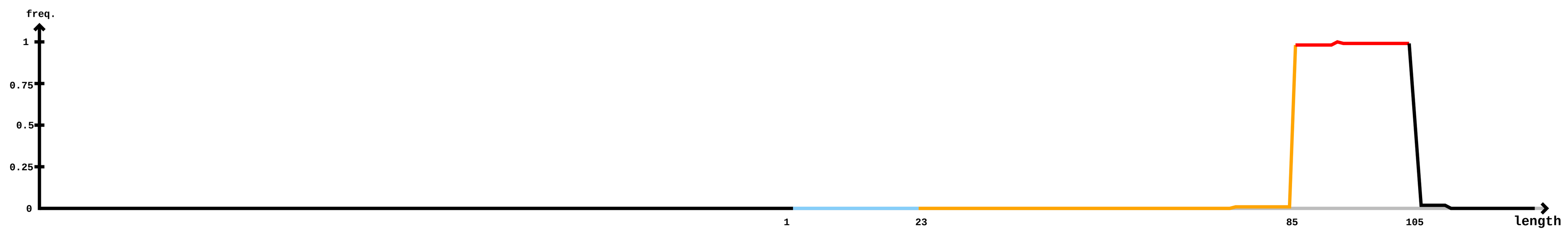

Star Mature

5'- nnnn…nnnnuuuuauuucccguuuuuuuuuuuuuuuuuuguuuuuuggugg cguuuuugugguuuguuuauuuauuuauucauucaucucccuauuuggauucugagaaaaaacugaaggaaauaaaguuuaaaacauuaacug -3' exp

((((((((((..(((..(((((((((...((((..(((.((.(.....((((..((..((......))..)).))))).)))))...))))...)))))))))..))))))))))))).................

sequence	reads	mm	sample
uauuuggauucugagaaa	1	0	LT-3
cugagaaaaaacugaaggaa	53	0	LCK-1
cugagaaaaaacuNaaggaa	1	1	LCK-1
cugagaaaaaacugaaggaa	49	0	LCK-2
cugagCaaaaacugaaggaa	1	1	LCK-2
aaaacugaaggaaauaaaU	2	1	LT-3
